# Identification of potential markers for differentiating epithelial ovarian cancer from ovarian low malignant potential tumors through integrated bioinformatics analysis

**DOI:** 10.1186/s13048-021-00794-0

**Published:** 2021-03-16

**Authors:** Wende Hao, Hongyu Zhao, Zhefeng Li, Jie Li, Jiahao Guo, Qi Chen, Yan Gao, Meng Ren, Xiaoting Zhao, Wentao Yue

**Affiliations:** Central Laboratory, Beijing Obstetrics and Gynecology Hospital, Capital Medical University, Beijing, 100026 China

**Keywords:** Epithelial ovarian cancer, Low malignant potential tumor, Integrated bioinformatical analysis, Chemicals, Diagnosis, Prognosis

## Abstract

**Background:**

Epithelial ovarian cancer (EOC), as a lethal malignancy in women, is often diagnosed as advanced stages. In contrast, intermediating between benign and malignant tumors, ovarian low malignant potential (LMP) tumors show a good prognosis. However, the differential diagnosis of the two diseases is not ideal, resulting in delays or unnecessary therapies. Therefore, unveiling the molecular differences between LMP and EOC may contribute to differential diagnosis and novel therapeutic and preventive policies development for EOC.

**Methods:**

In this study, three microarray data (GSE9899, GSE57477 and GSE27651) were used to explore the differentially expressed genes (DEGs) between LMP and EOC samples. Then, 5 genes were screened by protein–protein interaction (PPI) network, receiver operating characteristic (ROC), survival and Pearson correlation analysis. Meanwhile, chemical-core gene network construction was performed to identify the potential drugs or risk factors for EOC based on 5 core genes. Finally, we also identified the potential function of the 5 genes for EOC through pathway analysis.

**Results:**

Two hundred thirty-four DEGs were successfully screened, including 81 up-regulated genes and 153 down-regulated genes. Then, 5 core genes (CCNB1, KIF20A, ASPM, AURKA, and KIF23) were identified through PPI network analysis, ROC analysis, survival and Pearson correlation analysis, which show better diagnostic efficiency and higher prognostic value for EOC. Furthermore, NetworkAnalyst was used to identify top 15 chemicals that link with the 5 core genes. Among them, 11 chemicals were potential drugs and 4 chemicals were risk factors for EOC. Finally, we found that all 5 core genes mainly regulate EOC development via the cell cycle pathway by the bioinformatic analysis.

**Conclusion:**

Based on an integrated bioinformatic analysis, we identified potential biomarkers, risk factors and drugs for EOC, which may help to provide new ideas for EOC diagnosis, condition appraisal, prevention and treatment in future.

**Supplementary Information:**

The online version contains supplementary material available at 10.1186/s13048-021-00794-0.

## Introduction

Epithelial ovarian cancer (EOC) has been reported to be the common cause of death for gynecological cancer [1]. Moreover, most EOC cases were diagnosed as advanced due to their vague symptoms [2]. Despite improvements in surgery and other treatments, the therapeutic efficacy and prognosis of EOC patients with advanced stage still remain worse due to lack of early and effective detection methods [3].

Unlike EOC, LMP tumor is a unique epithelial subtype of ovarian tumor that intermediates between benign and malignant tumors [4]. Meanwhile, LMP tumor is also known as borderline malignant ovarian cancer due to lack invasion of the underlying stroma [5]. Thus, the prognosis of LMP and EOC differ considerably due to their different invasiveness, with 5-year survival rate > 90% for LMP versus a < 30% survival for advanced high-grade EOC [6]. Furthermore, unilateral oophorectomy should be considered in LMP patients in view of its younger onset age, which is different from EOC [7]. However, a portion of LMP tumors display diffuse non-invasive extra-ovarian implants, and accurate identification of these implants can be very difficult [4]. Moreover, approximately 20–30% of LMP cases are finally confirmed to be EOC [4]. Thus, it can be seen that the diagnosis based on histopathology without using molecular markers will lead to inaccurate diagnosis of LMP. Therefore, identifying potential differential diagnostic markers for LMP and EOC may improve the diagnostic accuracy and also contribute to the development of novel therapeutic & preventive strategies for EOC.

Recently, many integrated bioinformatical studies on EOC and normal samples have been shown to help explore the biomarkers and mechanisms of ovarian cancer occurrence and development [8]. However, current integrated bioinformatical studies on LMP and EOC may be insufficient.

In our study, DEGs between LMP and EOC were first screened based on three GEO datasets. Then, Kyoto Encyclopaedia of Genes and Genomes (KEGG) and Gene Ontology (GO) analyses were performed for these DEGs. Next, PPI network, ROC, survival and Pearson correlation analysis were utilized to further validate core genes, which show better diagnostic efficiency and higher prognostic value for EOC. Furthermore, the chemical-core gene network was constructed based on 5 core genes, and the top 15 related chemicals for EOC were identified. Finally, we found that all 5 core genes mainly regulate EOC development via the cell cycle pathway by the bioinformatic analysis, which may help to provide new ideas for EOC treatment.

## Materials and methods

### Data acquisition

Four gene expression profiles (GSE9899, GSE27651, GSE12172, and GSE57477) were downloaded from the Gene expression omnibus (GEO) database (http://www.ncbi.nlm.nih.gov/geo). GSE9899 included 18 ovarian LMP tumors and 267 EOC samples, GSE27651 included 8 LMP tumors and 22 EOC samples, GSE12172 comprised 30 LMP tumors and 60 EOC samples, and GSE57477 included 6 LMP tumors and 46 serous ovarian adenocarcinomas. The gene expression data of GSE9899, GSE27651 and GSE12172 were download from the platform of GPL570 (Affymetrix Human Genome U133 Plus 2.0 Array), whereas GSE57477’ gene expression data was download from the platform of GPL10558 (Illumina HumanHT-12 V4.0 expression beadchip).

### DEGs identification

The DEGs were identified between LMP tumors and EOC in GEO database by the Bioconductor package *limma* [9]. We set the |log_2_FC| > 1.0 and adjusted *P* < 0.05 for cutoff criteria. Then, the Venny online tool (https://bioinfogp.cnb.csic.es/tools/venny/) was applied to identify the overlapping DEGs among GSE9899, GSE57477 and GSE27651.

### Pathway analysis of DEGs

GO and KEGG analysis of DEGs were performed through the DAVID (https://david.ncifcrf.gov/). The top 20 items of GO function pathways and all items of KEGG pathways were then displayed as bubble diagrams using the *ggplot2* R package based on *P*-value (*P* < 0.05 as statistically significant).

### PPI and functional analysis

STRING (http://www.string-db.org/) was utilized to build PPI network of the identified DEGs [10]. Moreover, MCODE plugin from Cytoscape software (version 3.8.0) was performed to detect clusters of DEGs. Then, functional analysis of the genes in the hub module 1 were performed through the DAVID. Meanwhile, the top 20 hub genes were screened by the topological algorithm Maximal Clique Centrality (MCC) using the CytoHubba [11].

### Genetic information of the top 20 potential hub genes

The cBioPortal (https://www.cbioportal.org/) was utilized to show the genetic information of the top 20 hub genes.

### Validation of hub genes

The expression levels of the 20 genes between LMP tumors and EOC were verified through the GSE12172 dataset, and then the ROC and Kaplan–Meier analysis were used to explore their differential diagnostic and prognosis value. *P* < 0.05 was considered statistically significance.

### Pearson correlation analysis between 5 core genes’ expression and the level of different immune checkpoint proteins

Through the ROC and survival analysis, we screened the top 5 core genes with better diagnostic efficiency and higher prognostic value for EOC. We further analyzed whether 5 core genes’ expression is related to the level of different immune checkpoint proteins through the Gene Expression Profiling Interactive Analysis (GEPIA) database (http://gepia.cancer-pku.cn/detail.php) [12]. Then, the types of immune checkpoint proteins that are positively correlated with gene expression were selected and shown in picture.

### Chemical-core gene network analysis

Then, the network of the 5 core genes and their related chemicals was analyzed through the open-source platform NetworkAnalyst (https://www.networkanalyst.ca/) [13], and then visualized by Cytoscape software.

### KEGG and GO pathway analysis of the 5 core genes

The genes that were co-expressed with 5 core genes in EOC patients in the TCGA database were identified through cBioportal (http://cbioportal.org). Then, the co-expressed gene pairs with the Pearson correlation coefficient ≥ 0.3 were selected for further KEGG analysis through the DAVID. The top ten enriched KEGG terms for each core gene were shown in the form of matrix bubble map based on -log10(*P* value) through the community-driven bioinformatics data visualization platform Hiplot (https://hiplot.com.cn/basic/matrix-bubble). Meanwhile, GO biological process analysis for 5 core genes were also performed by the application of ClueGO [14] and CluePedia [15]. kappa coefficient of 0.42 and *P* < 0.05 were chosen as threshold values.

## Results

### DEGs identification

**Fig. 1:**
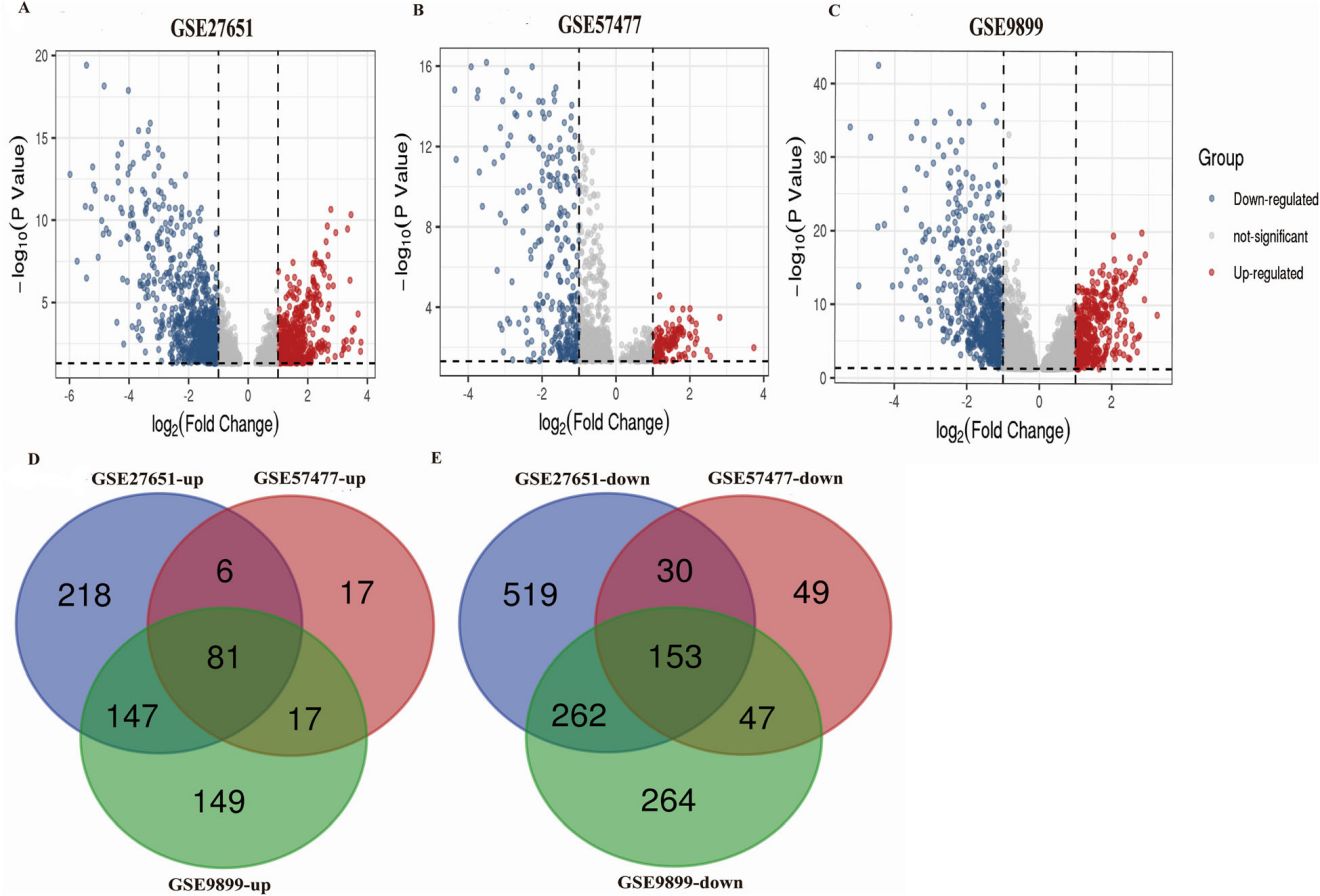
Overlapping DEGs Identification. Volcano plots of DEGs in GSE27651 (**a**) GSE57477 (**b**) and GSE9899 (**c**), respectively. Venn plots of up-regulated (**d**) and down-regulated (**e**) overlapping DEGs among GSE27651, GSE57477, and GSE9899 datasets

Screening criteria on the basis of adjusted P < 0.05 and |log_2_FC| > 1.0, DEGs were screened between LMP and EOC samples in GSE27651, GSE57477, and GSE9899. Then, the DEGs were visualized as volcano plots (Fig. 1a-c). Subsequently, 81 overlapping up-regulated genes and 153 down-regulated genes (Fig. 1d-e) were identified using Venny online tool.

### Pathway analysis

**Fig. 2:**
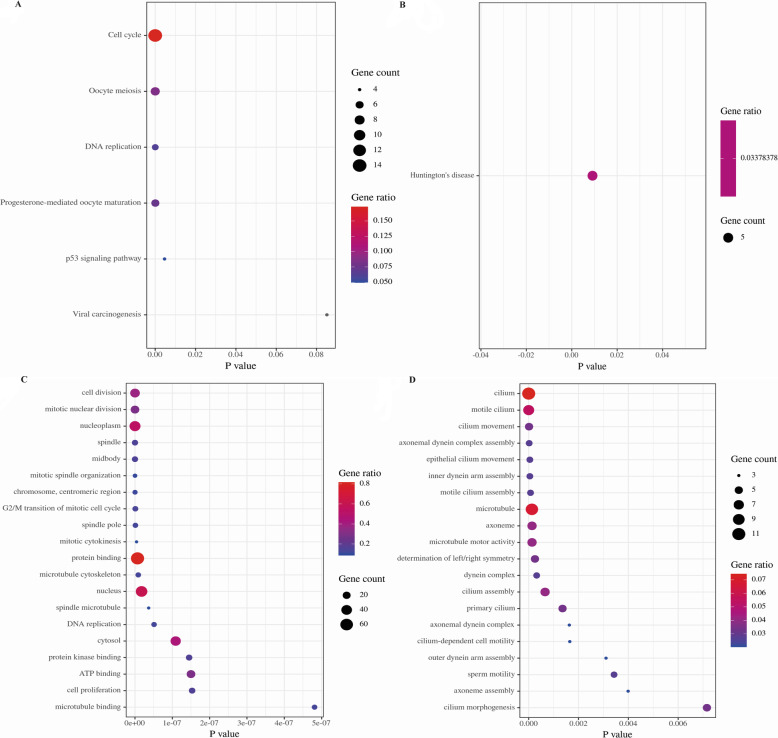
KEGG and GO analysis of overlapping DEGs. **a** KEGG analysis of up-regulated genes. **b** KEGG analysis of down-regulated genes. **c** GO analysis of up-regulated genes. **d** GO analysis of down-regulated genes

For a further understanding of the three datasets’ overlapping DEGs, KEGG and GO analysis were carried out through DAVID. In KEGG analysis, the overlapping up-regulated genes were highly enriched in Cell cycle and Oocyte meiosis (Fig. 2a), while the down-regulated genes were enriched in Huntington’s disease (Fig. 2b). In GO analysis, the overlapping up-regulated genes were highly enriched in Protein binding, Nucleoplasm and Nucleus (Fig. 2c), and the down-regulated genes were mostly enriched in Cilium, Microtubule, and Motile cilium (Fig. 2d).

### PPI network construction and cluster analysis

**Fig. 3:**
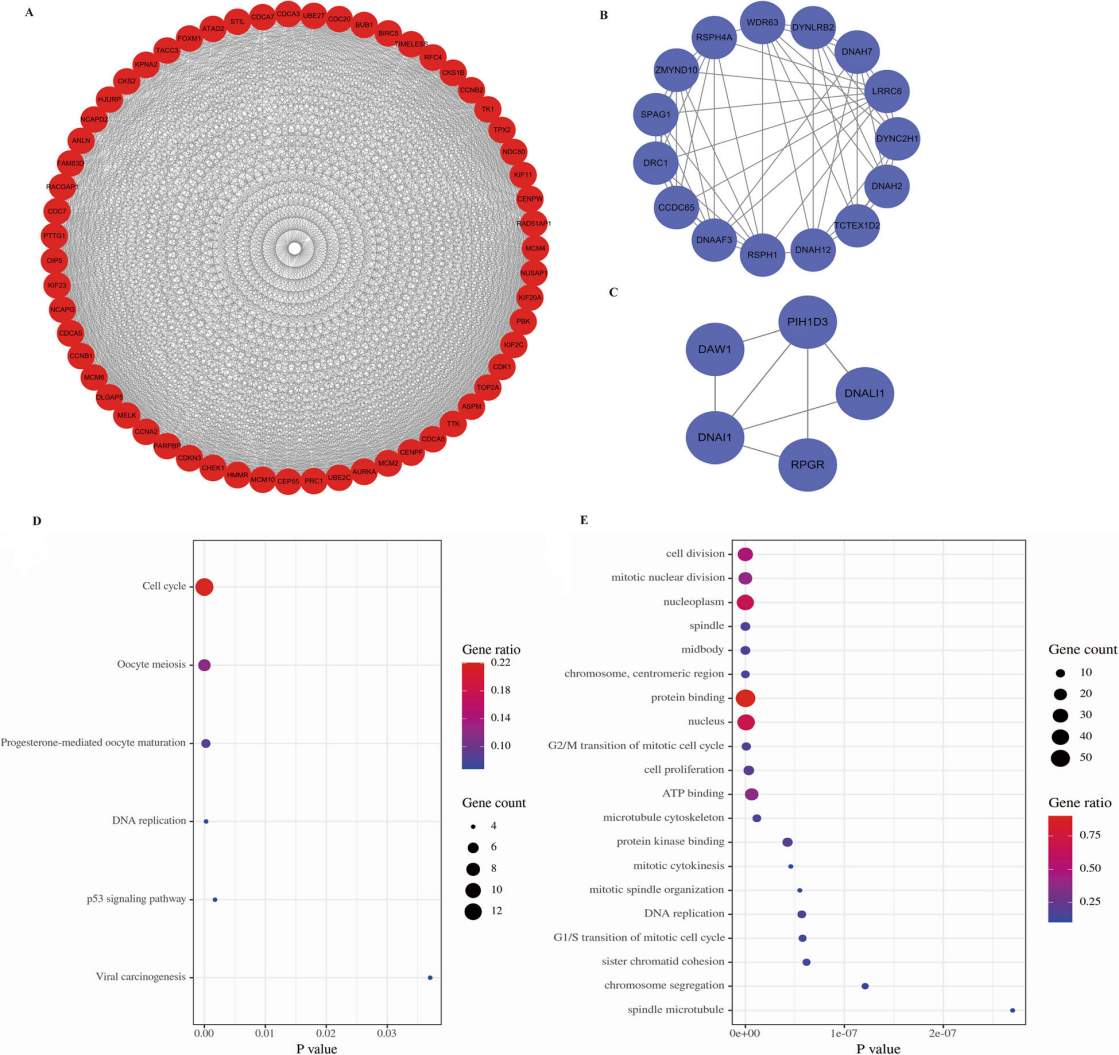
PPI network analysis. Cluster 1 (**a**), cluster 2 (**b**), cluster 3 (**c**). (note: red nodes represent up-regulated genes, while blue nodes represent down-regulated genes); **d** KEGG analysis of the genes in cluster 1. **e** GO analysis of the genes in cluster 1

The STRING database and the Cytoscape software were used to construct the PPI network of the overlapping DEGs in EOC. Finally, three important clusters were screened by MCODE. Among them, cluster 1 included 59 proteins with the highest score (Fig. 3a), cluster 2 and 3 contained 15 and 5 proteins, respectively (Fig. 3b & c). Additionally, we analyzed the function of cluster 1. In KEGG analysis, the cluster 1′ DEGs were mostly enriched in Cell cycle and Oocyte meiosis (Fig. 3d). In GO analysis, cluster 1′ DEGs were mostly enriched in Protein binding, Nucleoplasm and Nucleus (Fig. 3e).

### Hub gene identification and their genetic alteration information analysis

**Fig. 4:**
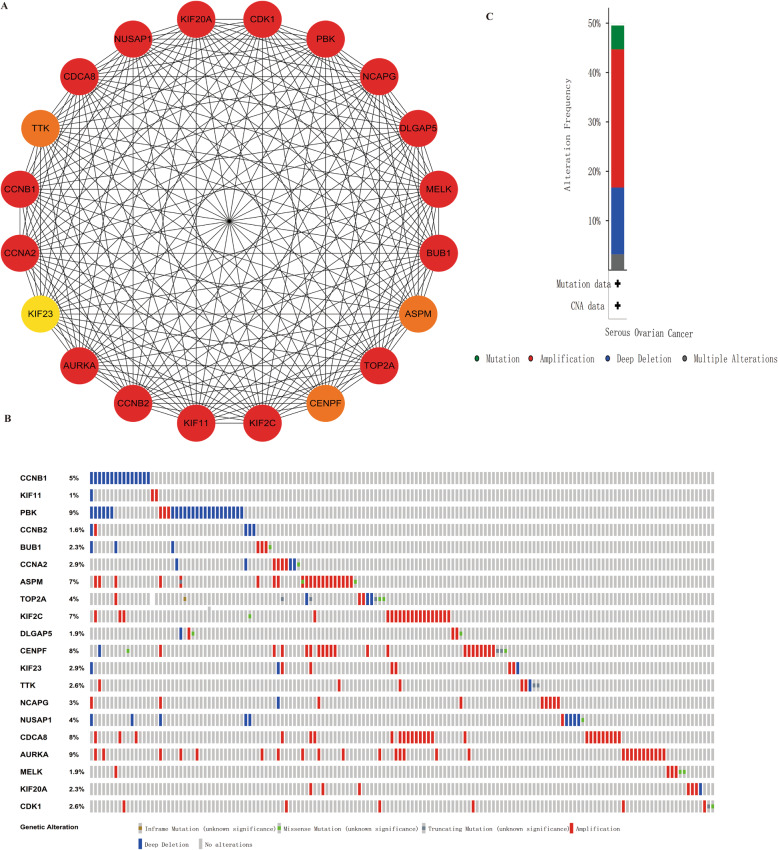
Hub genes and their genetic alterations identification. **a** The top 20 hub genes were identified through cytoHubba. (note: the redder the nodes color, the higher the ranking). **b** A visual summary of the hub genes’ genetic alterations in ovarian cancer samples. **c** An overview of the 20 genes’ genetic alterations in OV TCGA dataset

We further used the cytoHubba to identify hub genes in PPI network according to the topological algorithm maximal clique centrality (MCC). To identify more interested genes, we selected the top 20 genes for further analysis, which were also included in cluster 1(Fig. 4a and Table S1). Then, the genetic information of the 20 hub genes were shown through the cBioPortal (Fig. 4b & c). These genes were altered in 154 (50%) EOC samples or patients. PBK (9%) and AURKA (9%) were altered most frequently. Meanwhile, amplification accounted for the highest percentage of these alterations.

### Core gene validation

**Fig. 5:**
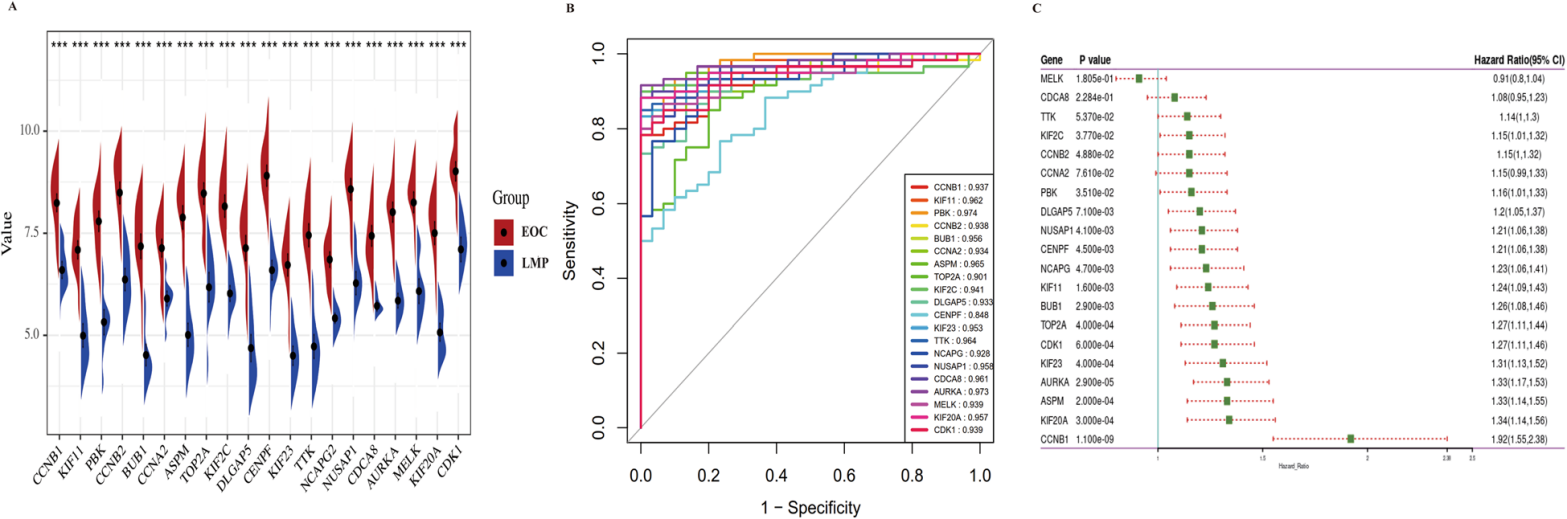
Hub gene validation. **a** The expression of 20 genes in LMP tumors and EOC samples in GSE12172. ***p < 0.001. **b** ROC curve analysis was performed to assess the capacity of 20 genes in differential diagnosis of LMP tumors and EOC. **c** Prognostic forest map of 20 genes in EOC patients

To further verify the role of the top 20 genes, we performed the expression analysis of LMP tumors versus EOC in GSE12172 dataset. The results showed that all 20 hub genes were higher in EOC (Fig. 5a). Meanwhile, ROC curve analysis was used to assess the capacity of these genes in differential diagnosis of LMP tumors and EOC in GSE12172, and almost all hub genes exhibited excellent diagnostic efficiency (AUC > 0.90) except for CENPF (AUC = 0.848) (Fig. 5b). Meanwhile, the prognostic value of the 20 genes for EOC was also assessed through the Kaplan-Meier plotter analysis. A prognostic forest map based on those genes is shown in Fig. 5c and 15 genes were significantly correlated to the overall survival (OS) of EOC patients. Moreover, EOC patients with higher levels of CCNB1 [HR = 1.92 (1.55–2.38), *P* = 1.1E-09], KIF20A [HR = 1.34 (1.14–1.56), *P* = 0.0003], ASPM [HR = 1.33 (1.14–1.55), *P* = 0.0002], AURKA [HR = 1.33 (1.17–1.53), *P* = 0.000029] and KIF23 [HR = 1.31 (1.13–1.52), *P* = 0.0004] were significantly related to worse OS.

### Pearson correlation analysis between 5 core genes and immune checkpoint proteins

**Fig. 6:**
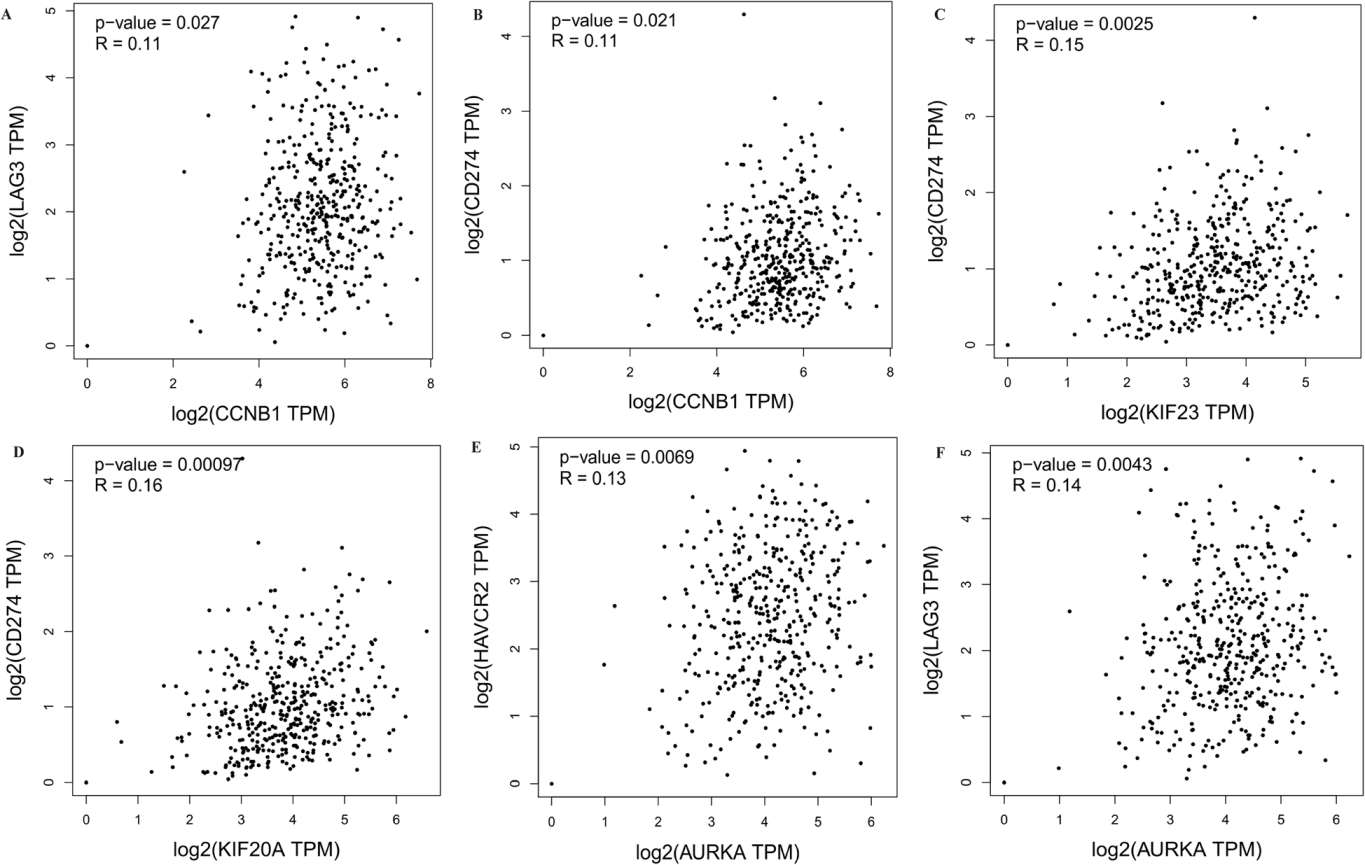
The positive correlation between the level of different immune checkpoint proteins and the expression of (**a** & **b**) CCNB1, (**c**) KIF23, (**d**) KIF20A, (**e** & **f**) AURKA in EOC

Through above analysis, we screened the top 5 core genes (including CCNB1, KIF20A, ASPM, AURKA, and KIF23) with better diagnostic efficiency and higher prognostic value for EOC. To further explore the role of the 5 core genes, we used the GEPIA to assess the correlation between gene expression and different immune checkpoint proteins (PD-L1, PD-1, CTLA-4, TIGIT, LAG3 and TIM-3) in EOC samples. As shown in Fig. 6, CCNB1 had positive correlation with the LAG3 (R = 0.11, *p* = 0.027) and PD-L1 expression (R = 0.11, *p* = 0.021) (Fig. 6a & b). KIF23 had positive correlation with PD-L1 expression (R = 0.15, *p* = 0.0025) (Fig. 6c). KIF20A had positive correlation with the PD-L1 expression (R = 0.16, *p* = 0.00097) (Fig. 6d). AURKA had positive correlation with the TIM-3 (R = 0.13, *p* = 0.0069) and LAG3 expression (R = 0.14, *p* = 0.0042) (Fig. 6e & f). The above results indicate that four in five core genes are closely related to the EOC immunosuppressive microenvironment, which may explain that they could be used as effective prognostic markers for EOC.

### Chemical-core gene network analysis

**Fig. 7:**
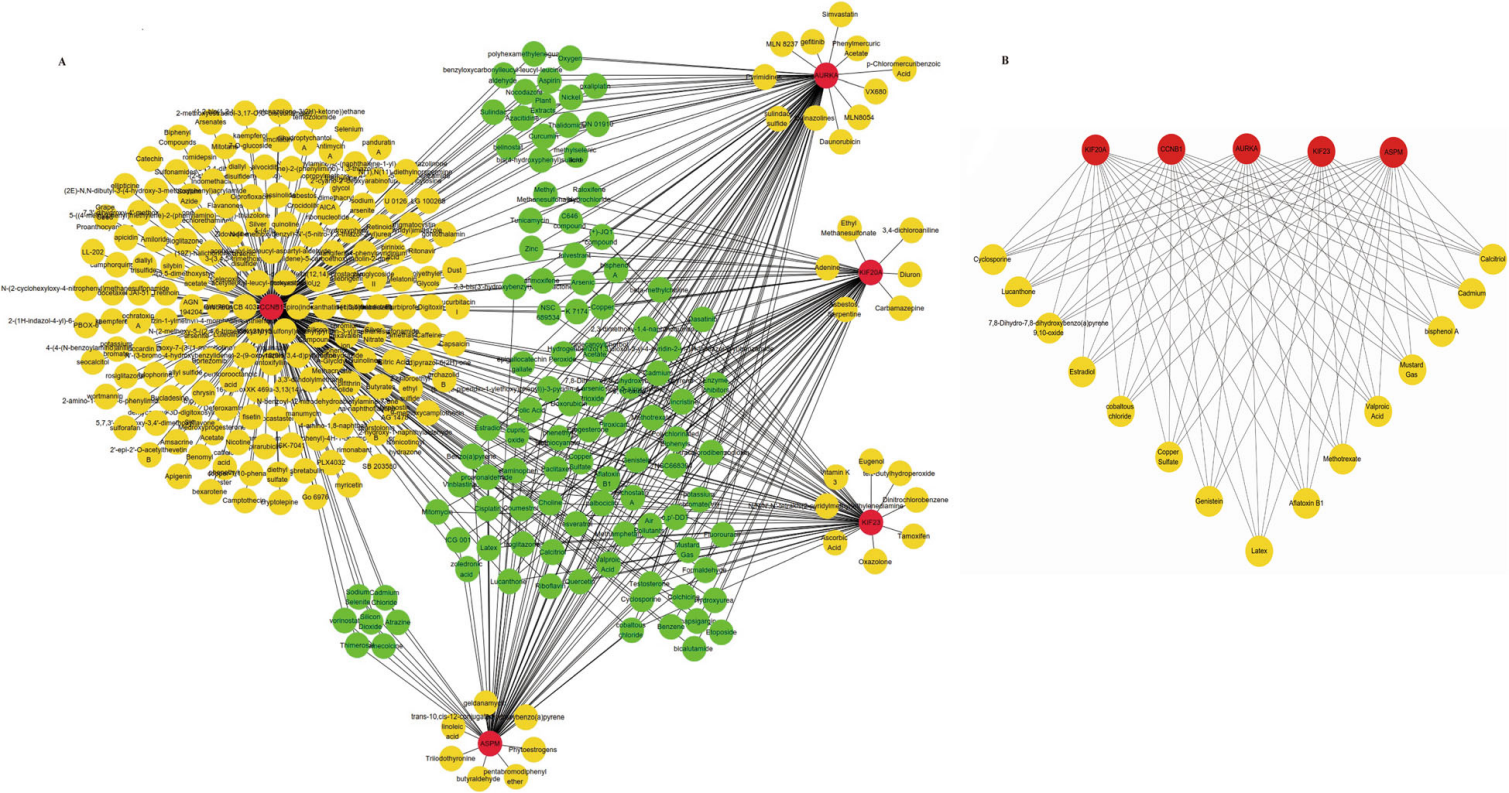
Chemical-core gene network analysis. **a** Interaction network between core genes and chemicals. **b** The top 15 chemicals screened by Cytoscape were related to all five core genes. Core genes are displayed in red nodes, whereas related chemicals are shown in yellow and green nodes

Then, NetworkAnalyst was used to screen the related chemicals for the 5 core genes. Meanwhile, the chemical-core gene network was drawn with the software of Cytoscape. As shown in Fig. 7a, the interaction network includes 5 core genes and 300 chemicals. Moreover, we found that most chemicals were related to CCNB1 (degree score = 246), AURKA (degree score = 86), and KIF20A (degree score = 67) (Table S2 and Fig. 7a), followed by ASPM (degree score = 63) and KIF23 (degree score = 52) via ranking the top 20 nodes in the network by cytoHubba. Furthermore, the top 15 chemicals screened by Cytoscape were found to be related with all five genes (Fig. 7b).

### KEGG and GO pathway analysis for 5 core genes

**Fig. 8:**
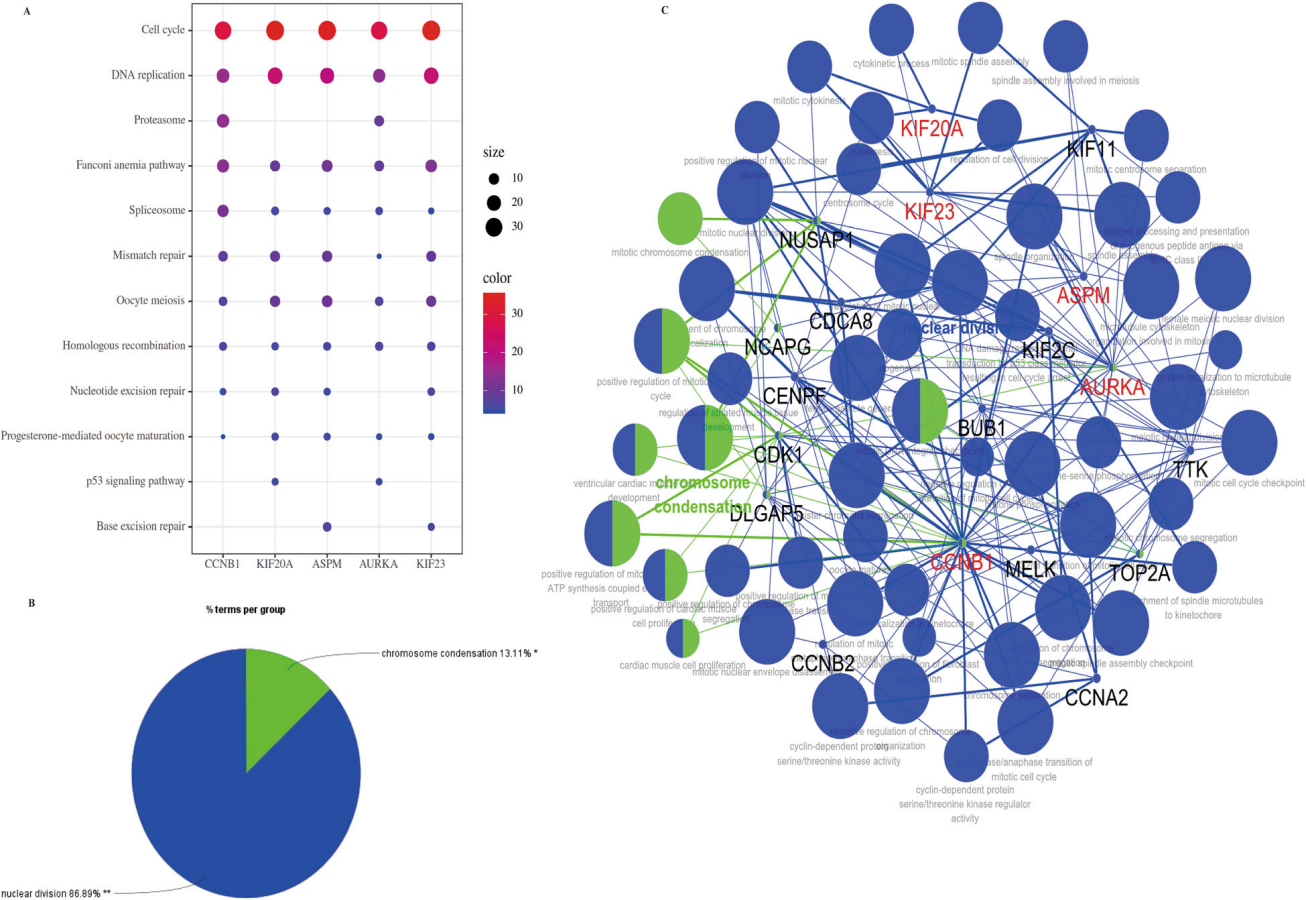
KEGG and GO analysis of 5 core genes. **a** KEGG analysis for 5 core genes in OC. **b** GO biological process analysis of specific gene cluster. **c** A network of the GO analysis by ClueGo and CluePedia (all 5 core genes were highlighted red)

We also identified the potential function of these 5 core genes in EOC through KEGG and GO pathway analysis. In KEGG pathway analysis, we observed that “Cell cycle” was the key player in all 5 core genes (Fig. 8a). Meanwhile, we investigated the previous 20 hub genes screened by cytoHubba for GO analysis by the application of ClueGO and CluePedia. As shown in Fig. 8b, all genes were involved in two different biological processes, including the nuclear division and chromosome condensation (Fig. 8b). Furthermore, we found that all 5 core genes were involved in nuclear division processes (Fig. 8c).

## Discussion

LMP, as a semi-malignant ovarian tumor, was identified by FIGO in 1971 and accounts for 15–20% of epithelial ovarian tumors [16, 17]. Moreover, LMP tumors often behave as a local epithelial lesions of the ovary [18]. By comparison, EOC often shows strong invasive characteristics and represents about 70% of epithelial ovarian tumors [6]. Thus, the prognosis of LMP and EOC differ considerably due to their different invasiveness. Additionally, the treatments of LMP and EOC are also signifi- cantly different. For EOC, total hysterectomy and bilateral salpingo-oophorectomy are the recommended surgical procedures and chemotherapy will also be administered to improve the curative effect [7]. But for LMP, hysterectomy is the standard therapy and unilateral oophorectomy will also be considered in view of the patients’ desire to maintain fertility [6]. However, although most LMP tumors will be in a mild state over a long period of time, a certain proportion of them display non-invasive extra-ovarian implants [19]. And the accurate identification of LMP tumors is a continuously challenging and controversial field in gynecological pathology due to difficulty in accurate classification of implants [20]. From this, accurate diagnosis of LMP and EOC is very important for the appropriate therapy selection and prognosis of patients, and the lack of accurate markers for distinguishing EOC from LMP will lead to incorrect diagnosis, inappropriate treatment and adverse consequences. In our study, we integrated three GEO databases and identified 234 DEGs between LMP and EOC samples, and further functional analysis was performed. The KEGG analysis displayed that the common up-regulated DEGs were mainly enriched in Cell cycle and Oocyte meiosis, while the common down-regulated genes were enriched in Huntington’s disease. In addition, the GO analysis showed that the common up-regulated DEGs were mainly associated with Protein binding, Nucleoplasm and Nucleus, while the common downregulated genes were mainly associated with Cilium, Microtubule, and Motile cilium. In fact, many studies have shown that the Cell cycle [21], Oocyte meiosis [22], Protein binding [23], Nucleoplasm [24], Nucleus [25], Microtubule [26], Cilium and Motile cilium [27] are closely related to EOC occurrence and development. However, the association between Huntington’s disease and EOC still remains unclear. In brief, these functional enrichment results have certain guiding significance. Furthermore, a PPI network analysis was performed for the DEGs, and then the MCODE plug-in filtered out three related clusters. We further analyzed the function of cluster 1 and found that the results are consistent with the previous analysis. Next, the top 20 genes in PPI network were identified by CytoHubba plug-in. Subsequently, the GSE12172 dataset was used to further verify their expression in LMP and EOC samples, and the results showed that all 20 genes were higher expressed in EOC compared with LMP tumors, meaning that these genes may play a key role in EOC tumorigenesis. Meanwhile, ROC curve analysis revealed that all these genes had perfect diagnostic efficiency for differentiating EOC from LMP tumors. In addition, survival analysis of these 20 genes showed that 5 genes (CCNB1, KIF20A, ASPM, AURKA, and KIF23) were significantly related to a poorer OS in EOC patients, which may be partly attributed to the tumor immunosuppressive microenvironment when performing the Pearson correlation analysis between 5 core genes’ expression and the level of different immune checkpoint proteins. Then, NetworkAnalyst was applied to screen the chemicals that were associated with the 5 core genes and we found that CCNB1, AURKA, and KIF20A were clearly the three interactive core genes that link most chemicals. Moreover, the top 15 chemicals screened by Cytoscape were found to be related with all 5 genes. Among them, valproic acid [28], Calcitriol [29], cobaltous chloride [30], Copper Sulfate [31], Genistein [32], 7,8-Dihydro-7,8-dihydroxybenzo(a) pyrene 9,10-oxide [33], Methotrexate [34], Mustard Gas [35] and Cyclosporine [36] all have been showed to have antitumor activity against EOC in vitro or vivo, whereas bisphenol A [37], cadmium [38], Aflatoxin B1 [39] and Estradiol [40] all have cancer-promoting activity in EOC. However, Lucanthone and Latex have not been studied in EOC till now despite they showed anti-cancer effects in other cancers [41]. Thus, further clinical trials and studies are needed to identify and explore their impact on EOC in future.

Next, the 5 core genes and their current researches in ovarian cancer were introduced as follows.

CCNB1, also called Cyclin B1, is an important mitotic cyclin and produce complexes with p34(cdc2), which play a role in cell cycle [42]. Meanwhile, CCNB1 has been shown to be over expressed in a variety of tumors, including EOC [43, 44], and many studies have also demonstrated that cyclin B1 is involved in the differentiation, proliferation, metastasis and chemoresistance of ovarian cancer cell [45, 46].

KIF20A, is a microtubule-associated motor protein localized to the Golgi apparatus that is required for cell cycle mitosis [47]. Until now, KIF20A has been reported to be a key gene in the progression of many tumors, such as prostate cancer, colorectal cancer, gastric cancer, et al. [48–50]. Recently, KIF20A has also been proved to promote the invasion and metastasis of EOC, and could be seen as a valuable chemoresistance and prognostic biomarker for EOC patients [51–53].

ASPM (abnormal spindle-like microcephaly associated) was originally seen as a centrosomal protein regulating neurogenesis [54]. In addition, ASPM is also known to regulate mitosis through controlling microtubule disassembly and is widely highly-expressed in a wide range of malignant tumors, including EOC [55–59].

AURKA, as a family of serine/threonine kinases, localizes in mitotic spindles and centrosomes where it mediates mitotic process and chromosome stability [60]. Mounting evidence have shown that AURKA is involved in the tumorigenesis and progression of multiple types of cancer including solid and hematological malignancies [61–63]. Meanwhile, a certain quantity of AURKA kinase inhibitors have been developed during the past decade, which suppress cancer cell proliferation, migration and invasion [61]. In our study, we identified several chemicals related with AURKA that against EOC, which may contribute to the development of novel potential drugs for the treatment of EOC.

KIF23, is a member of kinesin motor protein that regulates mitosis and cytokinesis [64]. KIF23 overexpression has been found in breast cancer, pancreatic cancer, primary lung cancer and prostate cancer, and also has been related with poor prognosis for several cancer types [65–68]. Recently, Tong Lia et al. has found that KIF23 was mainly related to cell cycle, and indicated a poor prognosis in EOC patients. Meanwhile, they also found that both miR-503-5p and miR-424-5p could directly targeted KIF23 to inhibit OC development in vitro [69]. Additionally, Hu Y et al. has reported that KIF23 could not only be used as a prognostic indicator for EOC, but also had a positive correlation with immune checkpoint protein, suggesting that it can be performed as a potential target for cancer immunotherapy, which is consistent with the results of our study [70].

Although above studies have found that these 5 genes related to the diagnosis, treatment, and prognosis of EOC, the molecular mechanisms of EOC tumorigenesis still remain unclear. In order to further understand the biological functions of these 5 genes in EOC, we performed the KEGG and GO pathway analysis for them. As a result, we observed that all 5 core genes were mainly enriched in “Cell cycle”, consistent with our previous pathway analysis of up-regulated DEGs in EOC. The results also demonstrate that all 5 core genes have potential value as targets of chemotherapy drugs for EOC, which is based on a universally accepted fact that most of the chemotherapeutic drugs are developed according to their regulation of cell cycle process.

## Conclusions

In brief, by performing an integrated bioinformatic analysis of three GEO datasets, we identified five core genes with better diagnostic efficiency and higher prognostic value for EOC and we also screened the potential drugs or risk factors for EOC through chemical-core gene network analysis base on them. Moreover, we found that all five core genes mainly regulate EOC development via the cell cycle pathway through the KEGG and GO pathway analysis. However, there are still a few limitations in our study. Since the study is based on data analysis, a large number of clinical samples and biological experiments are urgently needed to verify the results before promoting the clinical application of all five core genes as diagnostic and prognostic indicators or therapeutic targets in future.

## Supplementary Information

**Additional file 1: Table S1.** Top 20 hub genes screened by cytoHubba according to MCC.

**Additional file 2: Table S2.** Top 15 related chemicals screened by cytoHubba according to MCC.

## Data Availability

The datasets generated and analyzed during the current study are available from TCGA and GEO database.

## Abbreviations

EOC: Epithelial ovarian cancer
LMP: Ovarian low malignant potential tumors
GO: Gene Ontology
KEGG: Kyoto Encyclopedia of Gene and Genome
PPI: Protein–protein interaction
DEGs: Differentially expressed genes
MCODE: Molecular complex detection;
MCC: Maximal Clique Centrality
HR: Hazard ratio
OS: Overall survival
GEO: Gene expression omnibus
ROC: Receiver operating characteristic
TCGA: The Cancer Genome Atlas
FIGO: International Federation of Gynecology and Obstetrics
